# Association of Sleep Disorders With Physician Burnout

**DOI:** 10.1001/jamanetworkopen.2020.23256

**Published:** 2020-10-30

**Authors:** Matthew D. Weaver, Rebecca Robbins, Stuart F. Quan, Conor S. O’Brien, Natalie C. Viyaran, Charles A. Czeisler, Laura K. Barger

**Affiliations:** 1 Sleep Matters Initiative, Division of Sleep and Circadian Disorders, Departments of Medicine and Neurology, Brigham and Women’s Hospital, Boston, Massachusetts; 2 Division of Sleep Medicine, Harvard Medical School, Boston, Massachusetts

## Abstract

This cross-sectional study evaluates the association between sleep disorders and burnout symptoms among faculty and staff at a large teaching hospital.

## Introduction

Physicians’ mental health concerns affect the quality of life of caregivers, patient safety, health care expenditures, and occupational turnover. More than half of US physicians report burnout. Sleep deficiency is common—often a consequence of rotating or extended-duration shifts, night call, and competing demands. Sleep disturbance is a predictor of depression, and insufficient sleep may contribute to the development of burnout. Medical residents report that prolonged work hours negatively affect their quality of life. These factors suggest that sleep deficiency may be an underlying contributor to poor mental health in physicians.

We sought to identify the prevalence of sleep disorders and estimate the cross-sectional association between sleep disorders and burnout symptoms among faculty and staff in a large teaching hospital system.

## Methods

For this cross-sectional study, we developed a Sleep Health and Wellness (SHAW) program that was offered to hospital groups (eg, Anesthesiology, Orthopedics, and Radiology) through a series of 40-minute presentations during times typically reserved for grand rounds. The presentations were followed by tablet-based sleep disorder screening from May 2018 to May 2019. Participants received immediate screening results with an option to directly schedule sleep clinic appointments. Validated survey instruments were used to evaluate risk of obstructive sleep apnea,^1^ insomnia,^2^ restless legs syndrome,^3^ and shift work disorder.^4^ Burnout was assessed using the Maslach Burnout Inventory Human Services Survey. Consistent with prior work, we defined burnout as an emotional exhaustion score of 27 or higher and/or a depersonalization domain score of 10 or higher. Professional fulfillment was evaluated using the Professional Fulfillment Index.^5^ The association between sleep disorder screening status and burnout symptoms was tested using multivariable logistic regression models that controlled for group. These activities met institutional review board criteria for waiver of informed consent and were deemed exempt from institutional review board review by the Partners Human Research Committee because the activities were related to quality improvement.

## Results

One thousand four hundred thirty-six employees attended the SHAW program, and 1047 completed the sleep disorder screening (Table). More than a quarter of employees (306 [29%]) screened positive for at least 1 sleep disorder. The prevalence of sleep disorders varied across groups (maximum, 48%; minimum, 12%). The most common sleep disorder was insomnia (n = 140, 14%), followed by obstructive sleep apnea (n = 122, 12%), shift work disorder (n = 112, 11%), and restless legs syndrome (n = 26, 2%). In total, 58 (19%) of the 304 employees who had positive screening results for 1 or more sleep disorders scheduled an appointment during the session. Most (n = 280, 92%) of those who had a positive finding for a sleep disorder were previously undiagnosed and untreated.

**Table.** Number of Participants From Each Hospital Group^a^

Hospital group	Study participants, No. (%) (n = 1141)
Anesthesiology	64 (6)
Cardiovascular medicine	26 (2)
Dermatology	43 (4)
Emergency medicine	30 (3)
Endocrinology	37 (3)
Infectious disease	24 (2)
Internal medicine (continuing residents)	31 (3)
Internal medicine (new resident orientation)	80 (7)
Medical grand rounds (hospital A)	78 (7)
Medical grand rounds (hospital C)	23 (2)
Network medicine	67 (6)
Neurology (hospital A)	47 (4)
Neurology (hospital B)	42 (4)
Neurosurgery	47 (4)
Newborn medicine	38 (3)
Obstetrics and gynecology	38 (3)
Orthopedics	27 (2)
Pathology	41 (4)
Primary care	22 (2)
Separate primary care clinic	29 (3)
Pulmonary medicine	11 (1)
Radiation oncology	21 (2)
Radiology	65 (6)
Renal medicine	21 (2)
Rheumatology, immunology, allergy	28 (2)
Sleep medicine	50 (4)
Veteran’s Administration	51 (4)
Women’s health	60 (5)

^a^ In all, 1436 individuals attended, 1141 initiated the questionnaire, and 1047 completed the sleep disorder screening. This group information applies to the 1141 participants who initiated the questionnaire.

Of the 1074 employees who completed screening for burnout, 313 (29%) had a positive result. Of the 1031 employees who completed the professional fulfillment index, 508 (49%) reported low levels of professional fulfillment. The prevalence of burnout varied across groups (maximum 59%, minimum 10%). A positive sleep disorder screening result was associated with increased odds of burnout (odds ratio, 3.67; 95% CI, 2.75-4.89) and reduced odds of professional fulfillment (odds ratio, 0.53; 95% CI, 0.40-0.70) (Figure). Hospital group was not associated with burnout or professional fulfillment after adjustment for sleep disorder status.

**Figure.**
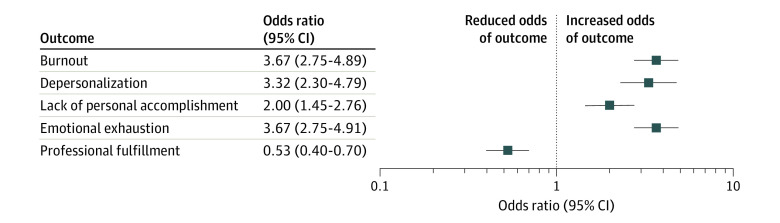
Association Between Sleep Disorder Screening and Occupational Burnout

## Discussion

The findings of this cross-sectional study suggest that undiagnosed sleep disorders are common among faculty and staff employed in a teaching hospital system. A positive screening result for a sleep disorder was associated with nearly 4-fold increased odds of occupational burnout. Those who had a positive screening result for a sleep disorder were half as likely to report professional fulfillment. More than 90% of sleep disorders were undiagnosed and untreated.

Treatment of sleep disorders may provide a novel means of intervening to reduce physician burnout, which has been resistant to other treatment approaches.^5^ We previously found that a similar sleep health and wellness program was effective in increasing the rates of evaluation, diagnosis, and treatment for sleep disorders among first responders.^6^ The current effort is limited by its cross-sectional design and limited collection of potential confounders. Further research is needed to determine whether facilitating treatment for common sleep disorders would reduce burnout in physicians.

This study suggests that undiagnosed and untreated sleep disorders are associated with occupational burnout among health care providers. Future studies should be conducted to evaluate the effectiveness of a sleep health and wellness program on reducing burnout symptoms.
